# Posterior cruciate ligament mediated avulsion fracture of the lateral tibial condyle: a case report

**DOI:** 10.1186/1749-799X-5-67

**Published:** 2010-09-08

**Authors:** Hiroyasu Ogawa, Hiroshi Sumi, Katsuji Shimizu

**Affiliations:** 1 Department of Orthopaedic Surgery Gifu University, Graduate School of Medicine, 1-1, Yanagido, Gifu, Gifu, 501-1194 Japan; 2 Department of Orthopaedic Surgery Sumi Memorial Hospital,2-1, Shirotori, Shirotori-cho, Gujo, Gifu, 501-5121 Japan

## Abstract

Avulsion fractures of the posterior cruciate ligament (PCL) are uncommon. On the basis of the site of damage of the PCL, hyperflexion, pretibial trauma, and hyperextension are proposed as mechanisms of PCL injuries. On the other hand, avulsion fractures of the tibial condyle are also rare. We report a PCL-mediated avulsion fracture of the lateral tibial condyle along with the tibial insertion of the PCL by extension-distraction force on the knee that has not been previously described in any study. This rare case may imply that application of an extension-distraction force to the PCL cause the avulsion fracture.

## Background

Avulsion fractures of the posterior cruciate ligament (PCL) are uncommon. A few mechanisms of PCL injuries have been proposed on the basis of the site of damage of the PCL [1]. The most common mechanism of avulsion fractures of the PCL at the tibial insertion is a dashboard injury, in which the knee is in a flexed position, and a posteriorly directed force is applied to the pretibial area [1]. Avulsion fractures of the tibial condyle are also rare [2,3]. The most common subset of avulsion fractures of the lateral tibial condyle is the Segond fracture. It is a small avulsion fracture of the proximal lateral tibial condyle that is induced by a force applied on the lateral capsule and the associated meniscotibial ligament [3]. Herein, we report a PCL-mediated avulsion fracture of the lateral tibial condyle along with the tibial insertion of the PCL by a mechanism that, to the best of our knowledge, has not been previously described in any study. Our patient and his family were informed that the data obtained in this case would be submitted for publication, and they consented to its publication.

## Case presentation

A 33-year-old male forestry worker sustained an injury to his right knee. While he was climbing a steep hill, a rolling log hit the anterior aspect of the extended knee at the level of the tibial tubercle with a posteroinferiorly directed force. At this time, his right lower leg was pulled down and extended forcibly by the rolling log, and he experienced immediate pain, swelling of the knee, and inability to bear weight on the right leg. The patient was transferred to our hospital for examination.

**Figure 1:**
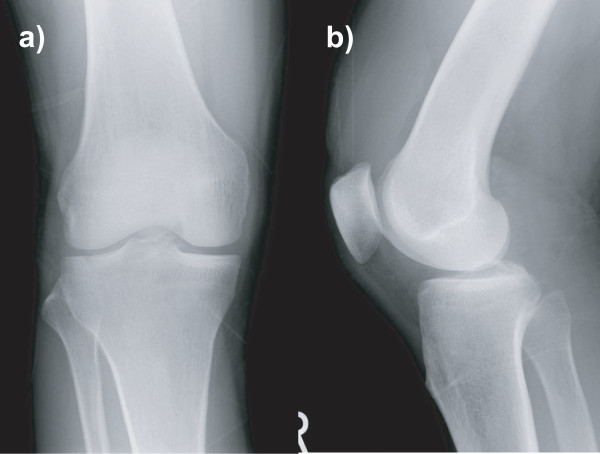
**Initial anteroposterior and lateral radiographs of the knee, showing fractures of the lateral tibial condyle (a) and the posterior intercondylar prominence (b)**.

**Figure 2:**
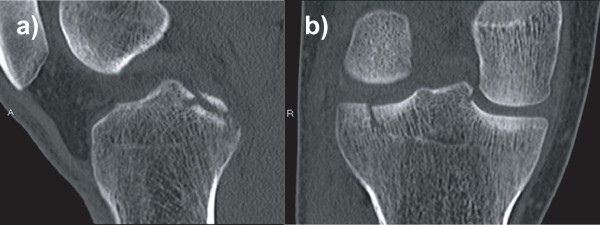
**Computed tomography scans showing sagittal and coronal views of the knee: the displaced fragments of the posterior intercondylar eminence (a) and the avulsed lateral tibial condyle (b) can be observed**.

**Figure 3:**
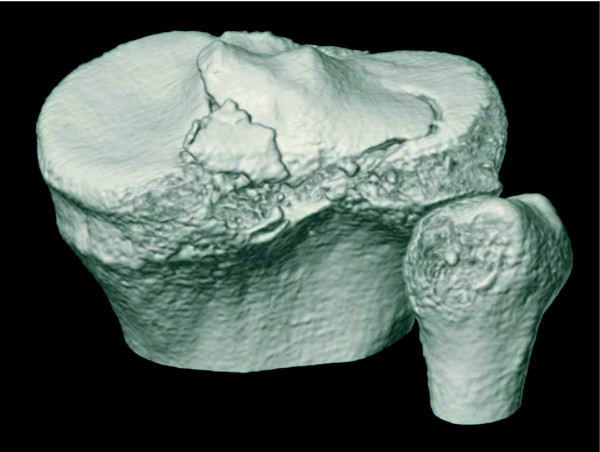
**Computed tomography scans showing three-dimensional reconstruction views of the knee: the displaced fragments of the posterior intercondylar eminence and the lateral tibial condyle can be observed**.

**Figure 4:**
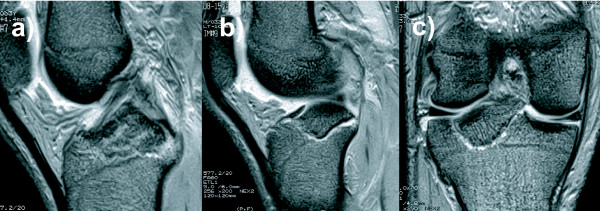
**Sagittal and coronal T2-weitghted magnetic resonance images of the knee, showing upward displacement of the PCL insertion at the tibia (a) and the displaced fragment of the lateral tibial condyle (b and c)**.

On clinical examination, we found that he had significant effusion in the affected knee. He complained of pain in the knee, especially during active and passive extension of the knee. There was tenderness at the posterior aspect of the knee, and no deficit was found in the neurovascular system. The knee was stable under varus and valgus stress, and the result of the anterior drawer test at 90° of knee flexion was negative. Posterior tibial sag was apparent, and the posterior drawer test with the knee flexed at 90° revealed a grade III instability (approximately 12 mm of posterior translation) with a soft endpoint. Radiographs revealed fractures of the posterior intercondylar eminence and the lateral tibial condyle (Fig. 1). The positions and sizes of the displaced fracture fragments were verified using computed tomography (CT) (Figs. 2 and 3). Magnetic resonance imaging (MRI) of the knee revealed that the anterior cruciate ligament (ACL), collateral ligaments, and both meniscuses were intact. The appearance of the posterior cruciate ligament (PCL) was consistent with that of a PCL avulsion fracture at the tibial insertion, and the lateral tibial condyle was avulsed anterosuperiorly without disruption of the articular cartilage (Fig. 4).

Arthroscopic examination revealed that the ACL, both meniscuses, collateral ligaments, and popliteal tendon were intact; the substance of the PCL was loose but not ruptured, and the cartilage surface of the lateral tibial condyle was not fractured but undulating. These findings were consistent with the MRI findings. On the basis of these findings, especially considering the PCL instability and his activity as a forestry worker, he was selected as a candidate for surgery.

During the operation, the patient was placed in a prone position, and a sinusoidal incision was made from the biceps femoris muscle to the medial head of the gastrocnemius muscle, with the transverse limb of the incision across the knee-flexion crease. The lesser saphenous vein and the medial sural cutaneous nerve were identified over the popliteal fascia, and these structures were protected with a Penrose drain. A longitudinal incision was made in the fascia along the margin of the medial sural cutaneous nerve in order to identify the tibial nerve, the popliteal artery and vein, and medial border of the medial head of the gastrocnemius. Continuation of the deep blunt dissection allowed the identification of the oblique popliteal ligament and the posterior capsule, which were found to be intact. A vertical incision was made across the capsule, and both edges of the incision were elevated. The avulsion fracture at the tibial insertion of the PCL was identified, and the lateral tibial condyle was also found to be anterosuperiorly avulsed. Neither meniscosynovial junction injuries nor compression injury to the lateral tibial condyle were observed. The fragment of the lateral tibial condyle, including the cartilage, was approximately 10-mm thick, and it formed approximately 40% of the articular surface of the lateral tibial condyle. These fragments were independent, but they shared a common bed. Furthermore, the fragment of the lateral tibial condyle partially formed the bed of the fragment of the tibial insertion of the PCL. After debridement of the base, the fragment of the lateral tibial condyle was reduced anatomically and stabilized using a Kirschner wire. Next, the fragment of the tibial insertion of the PCL was fitted into the crater, which was framed by the fragment of the lateral tibial condyle and the intercondylar eminence. After the fragments were stabilized with Kirschner wires, definitive fixation was achieved using a 3.5-mm cortical screw for the fragment of the tibial insertion of the PCL and a 4.0-mm cancellous screw for the fragment of the lateral tibial condyle. These screws were positioned from the posterosuperior to the anteroinferior side to achieve compression. The capsule, subcutaneous layers, and skin were subsequently closed. The knee was kept at 30° flexion in a cast.

**Figure 5:**
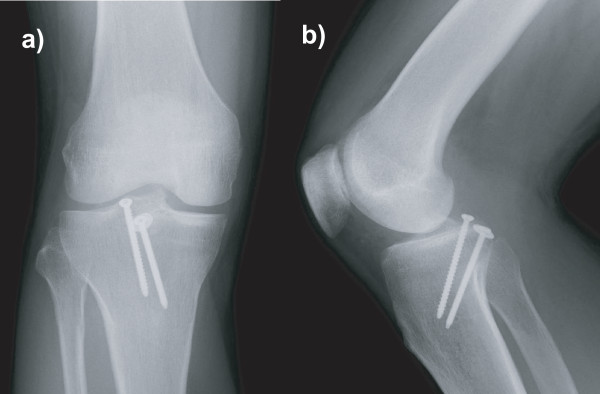
**Radiographs (a and b) at the 3-month follow-up showing union of the bone fragments**.

After the operation, the patient's leg was placed in a hinged knee brace fixed at at 30° flexion, the patient was permitted to bear partial body weight on the toes on postoperative day 1. Active flexion and extension exercises of the knee were initiated 2 weeks after the operation. At the 6-month postoperative follow-up examination, the patient's leg had regained its preoperative function with a full range of motion of the knee. Radiographs obtained 6 months after surgery also revealed healed fractures and no loss of fixation (Fig. 5), and the posterior drawer test revealed a grade-I instability (approximately 3 mm of posterior translation) with a hard endpoint.

## Discussion

This case report describes an unusual PCL-mediated avulsion fracture of the lateral tibial condyle. This injury may be different from other reported PCL injuries because it was caused by a mechanism that has not been reported previously, to the best of our knowledge.

In this case, a fragment of the lateral tibial condyle was avulsed anterosuperiorly along with the fragment of the tibial insertion of the PCL. The fragment of the lateral tibial condyle partially formed the base of the fragment of the tibial insertion of the PCL. These findings, in particular, the shape and position of the fragments, strongly suggested that the tibial insertion of the PCL was isolated from the fragment of the lateral tibial condyle subsequent to the PCL-mediated avulsion fracture of the lateral tibial condyle. The lack of comminution, joint depression, capsular injury, and visible cartilage damage also favored the role of an avulsion mechanism in the fracture of the lateral tibial condyle. Avulsion fractures of the posterolateral tibial condyle are rare because there is no muscle insertion on the posterior aspect of the lateral tibial condyle. This unusual injury pattern may be due to the manner in which the injury is suffered and the anatomy of the PCL, which should be investigated to elucidate the fundamental functions of the PCL.

PCL injuries usually occur at the femoral origin, in the substance, and at the tibial insertion of the PCL [4]. However, in this case, the main injury site was the lateral tibial condyle. The lateral tibial condyle is rarely damaged as a result of PCL injuries, though damage to the lateral condyle may be broadly categorized as a tibial insertion site injury. Depending on the site that has been damaged, 3 possible mechanisms for PCL injuries have been proposed as follows [1]. (1) Hyperflexion is commonly observed in individuals involved in sport activities. In this case, the PCL is guillotined between the posterior tibial plateau and the roof of the femoral notch, resulting in rupture of the midsubstance [5]. (2) Dashboard injury is a common injury observed in pretibial trauma, in which the knee is in a flexed position and a posteriorly directed force is applied to the pretibial area. This type of an injury results in a substance tear at the level of the tibial plateau or a tibial avulsion fracture [6]. (3) Hyperextension can result in proximal rupture of the PCL and posterior capsule. PCL injuries frequently occur as proximal disruption of the femoral attachment [7]. However, the mechanism of injury in this case did not correspond to any of the above mechanisms from the perspective of the relationship of the mechanism to the site of damage. This is because the knee extension that occurred in the case of our patient was inconsistent with a pretibial trauma, and the lack of a posterior capsule tear and soft tissue damage were inconsistent with a hyperextension injury. Furthermore, a recent biomechanical study concerning knee hyperextension revealed that knee hyperextension showed a general injury pattern to the posteolateral corner and no gross posterior cruciate ligament injuries [8], which may contradict simple hyperextension mechanism in this case. An in-depth interview of the patient revealed that the injury might have been caused by extension-distraction of the knee when the log rolling down a steep hill hit the extended knee with a posteroinferiorly directed force though we could not still neglect posteriorly directed force on the extended knee at the level of the tibial tubercle. Subsequently, the extension-distraction force may have acted on the PCL only to a slight extent so that the lateral tibial condyle was avulsed, but the posterior capsule was not injured. Hyperextension would lengthen all structures posterior to the knee axis, but mechanically, this lengthening force would be greater more posterior to the knee axis. Thus, an independent hyperextension force would lengthen the posterior capsule to a greater extent than the PCL, which would predispose the posterior capsule to rupture. In contrast, a distraction force would lengthen all the structures in the knee, and this force would be greater in tighter and shorter structures, which may result in lengthening of only the PCL but not the posterior capsule. Thus, from the mechanical viewpoint and the radiographic and intraoperative findings, we consider the distraction force to be more dominant than the hyperextension force in this case because the PCL was injured but not the posterior capsule.

The morphology of the tibial insertion site of the PCL may be another cause of this specific PCL injury. The PCL separates into the anterolateral and posteromedial bundles [4,9]. The substances of these bundles vary in width. The surface areas of the tibial insertion sites also vary in size: the largest surface areas are 2 times larger than the smallest surface areas. However, the shapes and positions of these insertion sites are consistent in osseous landmarks. The PCL is attached to the posterior intercondylar fossa between the tibial plateaus, and it also extends below the posterior part of the tibial rim. This fossa is trapezoid in shape, and it widens inferiorly. Peripheral fibers of the PCL are attached extensively to the distal tibial periosteum [4]. Thus, the PCL can tolerate a distraction force that is strong enough to avulse the lateral tibial condyle if the surface area of the tibial insertion site and the substance of the PCL are significantly wider.

With regard to the treatment, nonoperative treatment was another choice, but loss of the range of motion of the knee with some residual PCL laxity can be a significant problem in long-term [10]. Furthermore, osteonecrosis or nonunion of the fragment of the tibial insertion of the PCL was another concern because a part of its base was framed by the fragment of the lateral tibial condyle; this was disadvantageous to blood supply. However, the open posterior approach allowed remarkable direct visualization of the fracture sites, and adequate reduction and fixation of the fragments could be successfully performed. In our opinion, the posterior approach was a better alternative than arthroscopic surgery in this case because of the complex fracture pattern of the tibial insertion of the PCL, which was revealed by radiographic and intraoperative findings. Preoperative three-dimensional reconstruction CT and MRI were performed to decide the surgical approach and the method for fixing the fragments.

PCL injury patterns are complex and are related to diverse mechanisms of injury and to the structure of the PCL. The major functions of the PCL are to resist posterior tibial translation, varus and valgus forces applied to the knee, as well as external rotation of the tibia [9]. However, the PCL is not known to resist a distraction force. This rare case may imply that application of an extension-distraction force to the PCL may cause avulsion fracture of the lateral tibial condyle. A biomechanical study is required to verify this function of the PCL against an extension-distraction force in the knee. This data obtained in this study and such biomechanical studies would be of value not only in further understanding the unique mechanism of PCL injuries but also the basic function of the PCL, which would help improve the methods used for reconstruction surgery of the PCL.

## Consent

Written informed consent was obtained from the patients for publication of this case report and the accompanying images and coupes.

## Competing interests

The authors declare that they have no competing interests.

## Authors' contributions

HO performed surgical procedure, designed manuscript, and collected patient information. HS participated in surgery and follow-up. KS advised on design of this report. All authors read and approved the final manuscript.
